# Clonal Changes in the Pneumococcal Population Carried by Portuguese Children during Six Years of Private Use of the 13-Valent Pneumococcal Conjugate Vaccine: the Relative Contribution of Clonal Expansion, Clonal Emergence, and Capsular Switch Events

**DOI:** 10.1128/spectrum.02909-22

**Published:** 2023-03-22

**Authors:** Catarina Candeias, Sofia Félix, Sara Handem, Hermínia de Lencastre, Raquel Sá-Leão

**Affiliations:** a Laboratory of Molecular Microbiology of Human Pathogens, Instituto de Tecnologia Química e Biológica António Xavier, Universidade Nova de Lisboa (ITQB NOVA), Oeiras, Portugal; b Laboratory of Molecular Genetics, ITQB NOVA, Oeiras, Portugal; c Laboratory of Microbiology and Infectious Diseases, The Rockefeller University, New York, New York, USA; UJF-Grenoble 1, CHU Grenoble

**Keywords:** MLST, serotype, *Streptococcus pneumoniae*, vaccine

## Abstract

In Portugal, the 13-valent pneumococcal conjugate vaccine (PCV13) was available for private use from 2010 to 2015 and it was introduced in the National Immunization Program in 2015. We have reported that private use of PCV13 led to extensive serotype replacement and an increase in antimicrobial susceptibility among pneumococci carried by healthy children. We investigated which clonal changes concurred with these observations. A total of 657 pneumococcal strains, representative of a collection of 2,615 isolates, were genotyped by multilocus sequence typing (MLST). The isolates were recovered in 2009 to 2010 (pre-PCV13), 2011 to 2012 (early PCV13), and 2015 to 2016 (late PCV13) from children attending day care centers in two regions of Portugal (one urban, one rural). One-hundred seventy-one sequence types (STs) were identified, corresponding to 18 clonal complexes (CCs) and 58 singletons. Most CCs (*n* = 17) and several singletons (*n* = 16) were found in both regions, indicating that they were geographically disseminated. Clonal complexes expressing PCV13 serotypes in circulation in the late PCV13 period were a subset of the ones identified in the pre-PCV13 period and were often associated with antimicrobial resistance. Among those, the most frequent in both regions was CC179, a multidrug-resistant clone of serotype 19F. Serotype replacement, following PCV13 use, was mainly due to expansion of the susceptible lineages expressing non-PCV13 serotypes already in circulation in the pre-PCV13 period. The emergence of ST53, associated with serotype 8, a major cause of disease in several European countries, was observed in the rural region. Potential capsular switching events, unrelated to PCV13 use, were detected. This study improves our understanding of changes triggered by the private use of PCV13 in Portugal.

**IMPORTANCE** Streptococcus pneumoniae (pneumococcus) is a major human respiratory pathogen linked with high morbidity, mortality, and health care-associated costs worldwide. This bacterium often colonizes asymptomatically healthy children. Colonization is a prerequisite for disease and is also essential for transmission between individuals. The 13-valent pneumococcal conjugate vaccine targets 13 of 101 capsular types of pneumococci described to date. This vaccine not only prevents pneumococcal disease but also impacts colonization by decreasing the carriage of vaccine serotypes. Consequently, serotype replacement occurs. The clonal changes occurring during serotype replacement may be due to various mechanisms, such as clonal expansion, emergence, extinction, or capsular switch (vaccine escape). This study shows that in Portugal, the use of PCV13 has led to significant changes in clonal composition and that these were mainly due to the clonal expansion of lineages expressing serotypes not included in the vaccine.

## INTRODUCTION

Streptococcus pneumoniae (pneumococcus) is a major human respiratory pathogen linked with high morbidity, mortality, and health care-associated costs worldwide. Pneumococcal disease can have several forms, such as otitis media, pneumonia, bacteremia, and meningitis. The main risk groups for pneumococcal disease are young children, the elderly, and immunocompromised patients (1). The natural lifestyle of the pneumococcus, however, is asymptomatic colonization of the upper respiratory tract of healthy people (2). Children frequently carry pneumococci in the nasopharynx and are considered the main reservoir (3). In Portugal, most children attend day care centers, and several studies have shown that on average, 60% to 65% of them carry pneumococci (4–7).

To overcome the high pneumococcal disease burden, vaccination strategies have been implemented over time, using the polysaccharide capsule as the primary target (8). In Portugal, the 13-valent pneumococcal conjugate vaccine (PCV13), targeting 13 serotypes (1, 3, 4, 5, 6A, 6B, 7F, 9V, 14, 18C, 19A, 19F, and 23F) of the 101 described to date (9), became available for private use in January 2010. PCV13 replaced PCV7 (a 7-valent pneumococcal conjugate vaccine targeting serotypes 4, 6B, 9V, 14, 18C, 19F, and 23F introduced one decade earlier). In July 2015, PCV13 was the first PCV to be introduced into the Portuguese National Immunization Program (NIP) (International Vaccines Access Center; https://view-hub.org/). Private use of PCVs prior to that point, however, was high (reaching 70% to 90%), despite the lack of reimbursement by the Portuguese state (4).

The use of PCVs has decreased the incidence of pneumococcal disease and the prevalence of colonization by vaccine types among vaccinated individuals. As some of the serotypes targeted by PCVs were associated with antimicrobial resistance, the use of PCVs was frequently accompanied by a decrease in antimicrobial resistance rates among pneumococci (10, 11). Concomitantly, serotype replacement with serotypes not targeted by PCVs was observed in colonization, and in some countries, this was also observed to have variable impact on the incidence of pneumococcal disease (4, 12–14).

We recently reported the impact of the private use of PCV13 in Portugal on pneumococci carried by children (4). Between 2009 to 2010 and 2015 to 2016, we observed extensive serotype replacement and an increase in antimicrobial susceptibility. In this study, we used multilocus sequence typing (MLST) to investigate clonal changes that occurred during this time and unveil the relative contribution of clonal expansion, extinction, emergence, and capsular switch events to the serotype changes previously documented.

## RESULTS

### Clonal relationships of pneumococcal carriage isolates.

**FIG 1:**
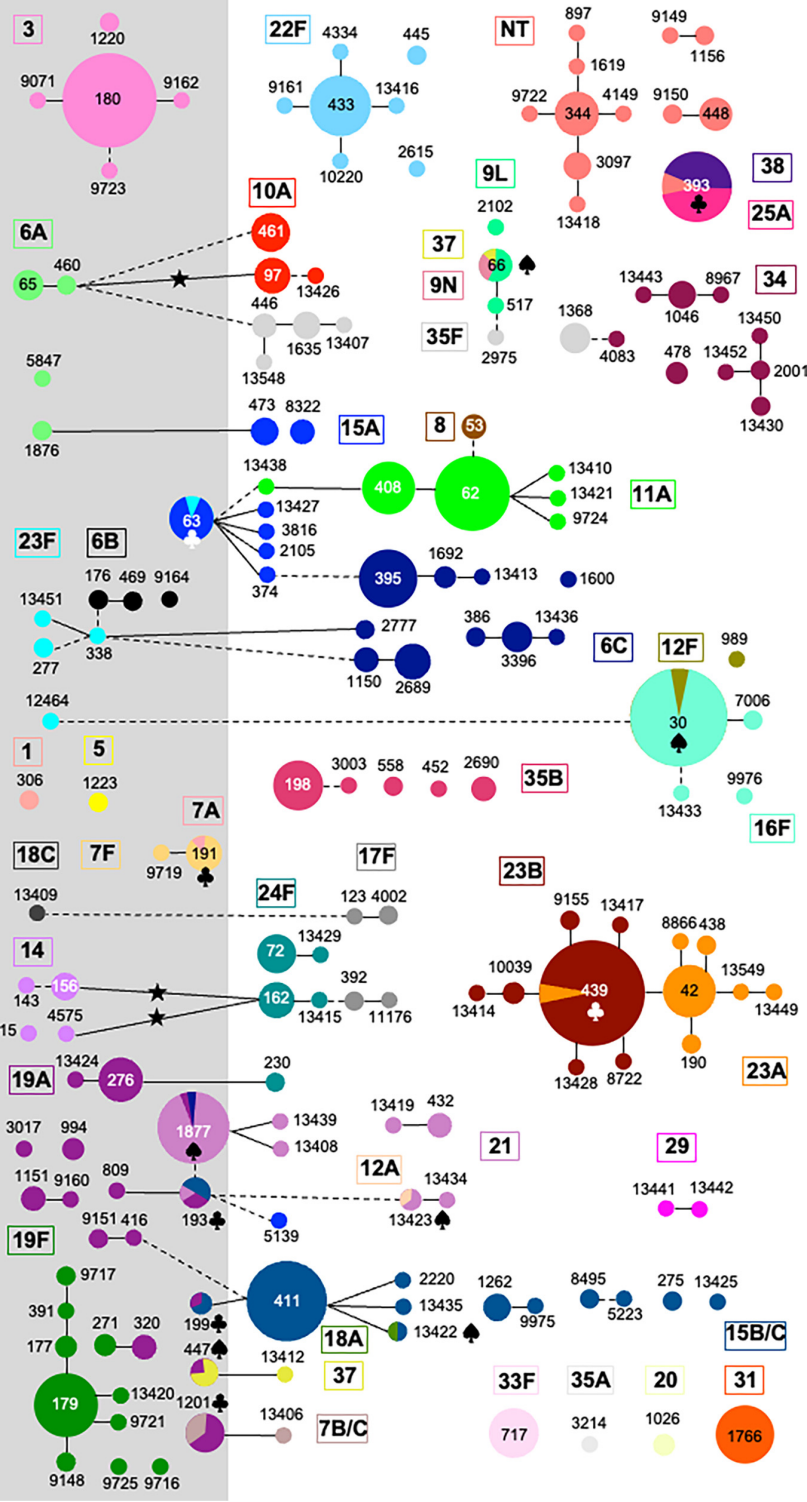
Clonal lineages of pneumococcal carriage isolates (2009 to 2016). Each circle represents one ST (indicated inside or near the circle); the size of the circle is proportional to the number of isolates. Each color represents a serotype (indicated inside the rectangles). Solid lines indicate single-locus variants (SLVs), and dotted lines indicate double-locus variants (DLVs); the length of the line does not have any meaning. The gray area indicates STs associated with PCV13 serotypes. Clubs indicate capsular switch events previously described; spades indicate capsular switch events described for the first time in this study. Stars indicate SLVs of STs previously associated with a PCV13 serotype but expressing a non-PCV13 serotype in the late PCV13 period. The schematic representation of the relationship between isolates was initially obtained using PHYLOViZ software with the goeBURST algorithm. The image was edited using PowerPoint software while maintaining the relationships obtained.

A total of 657 isolates representative of the pneumococci isolated in the pre-PCV13, early PCV13, and late PCV13 periods from the urban and rural regions were characterized using MLST (see Table S1 in the supplemental material). One-hundred and seventy-one STs were identified, among which there were four new alleles (*spi-582*, *gki-601*, *gki-602*, and *recP-418*) and 39 novel allelic combinations (corresponding to ST13406 to ST13410, ST13412 to ST13430, ST13433 to ST13436, ST13438, ST13439, ST13441 to ST13443, ST13449 to ST13452, ST13548, and ST13549) (Fig. 1). Ninety-one isolates (13.9%) had STs characteristic of the Pneumococcal Molecular Epidemiology Network (PMEN) clones: Netherlands^3^-180 (*n* = 28), Norway^NT^-344 (*n* = 11), Sweden^15A^-63 (*n* = 11), Netherlands^7F^-191 (*n* = 8), USA^NT^-448 (*n* = 7), Greece^21^-193 (*n* = 6), Spain^9V^-156 (serotype 14 variant; *n* = 5), Netherlands^8^-53 (*n* = 4), Netherlands^15B^-199 (*n* = 3), Portugal^19F^-177 (*n* = 3), Sweden^1^-306 (*n* = 2), Denmark^14^-230 (serotype 24 variant; *n* = 2), and Colombia^23F^-338 (*n* = 1). PMEN clones are characterized by having a large geographic distribution and by being epidemiologically relevant (15).

After goeBURST analysis, the 171 STs clustered into 18 clonal complexes (CCs) and 58 singletons (Fig. 1; Table S2). The number of isolates per CC ranged from 4 (CC123) to 77 (CC62) isolates; the number of isolates per singleton ranged from 1 (27 different STs) to 19 isolates (ST393). All except one CC were identified in both regions (CC123 was identified in the rural region only); 16 singletons were found in both regions, 24 in the urban region only, and 18 in the rural region only.

### Evolution of clones among PCV13 serotypes.

**FIG 2:**
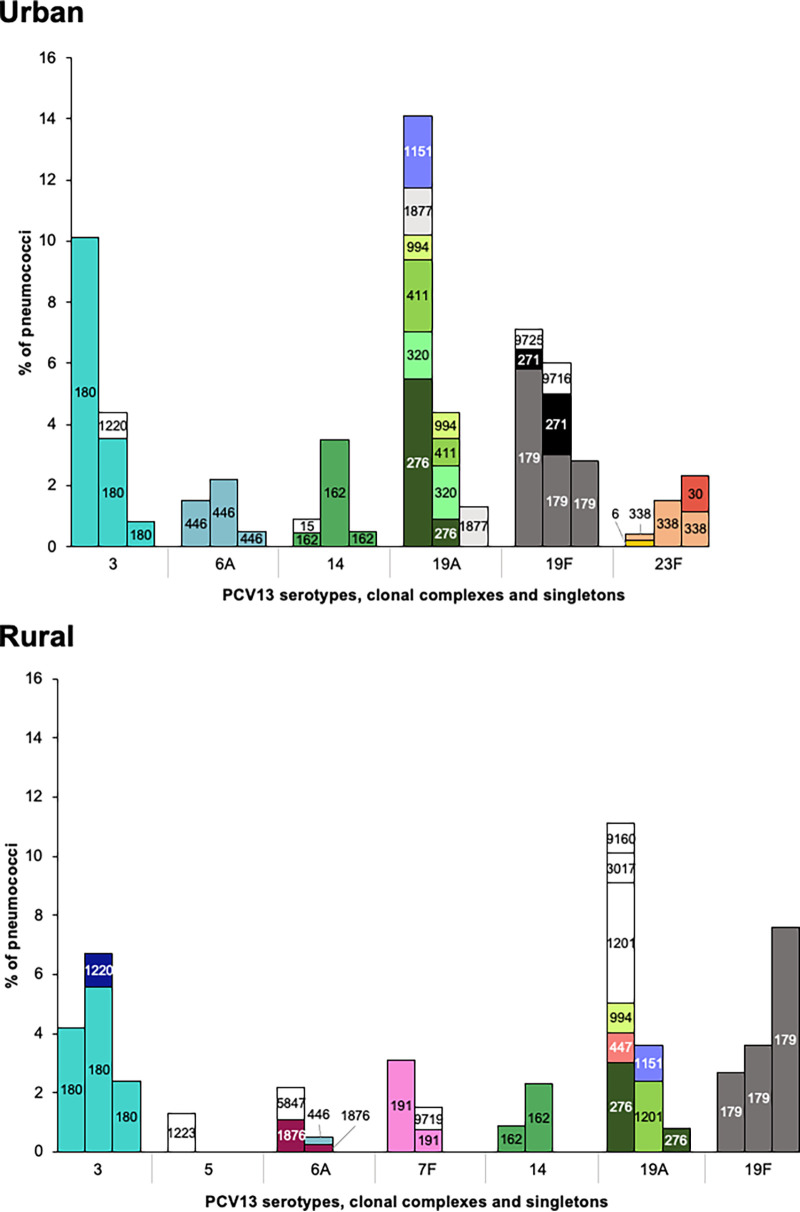
Clonal evolution of PCV13 serotypes over time in two regions. Each CC is shown in a different color, and singletons are depicted in white. The numbers inside the bars indicate the founding ST of each CC, as determined using goeBURST. The *y* axis represents the serotype-specific prevalence among pneumococcal carriers. For each serotype, the bars correspond to the pre-, early, and late PCV13 periods, from left to right, respectively. PCV13 serotypes not indicated were either not detected or only sporadically present.

We recently reported, in agreement with other studies, that upon the introduction of PCV13, the prevalence of PCV13 serotypes declined significantly in our population. Still, in the late PCV13 period, isolates of eight PCV13 serotypes (3, 6A, 6B, 14, 18C, 19A, 19F, and 23F) were detected (4). We investigated whether there had been significant clonal changes associated with this decline. We observed that in parallel with the reduction in the number of isolates, there was a reduction in the number of CCs associated with PCV13 serotypes in both regions (Fig. 1 and 2; Table S3). In the late PCV13 period, the 20 isolates analyzed using MLST (11 from the urban region and 9 from the rural region) belonged mostly (*n* = 18) to clonal complexes detected in previous periods (Fig. 1 and 2; Table S3).

### Evolution of clones among non-PCV13 serotypes.

**FIG 3:**
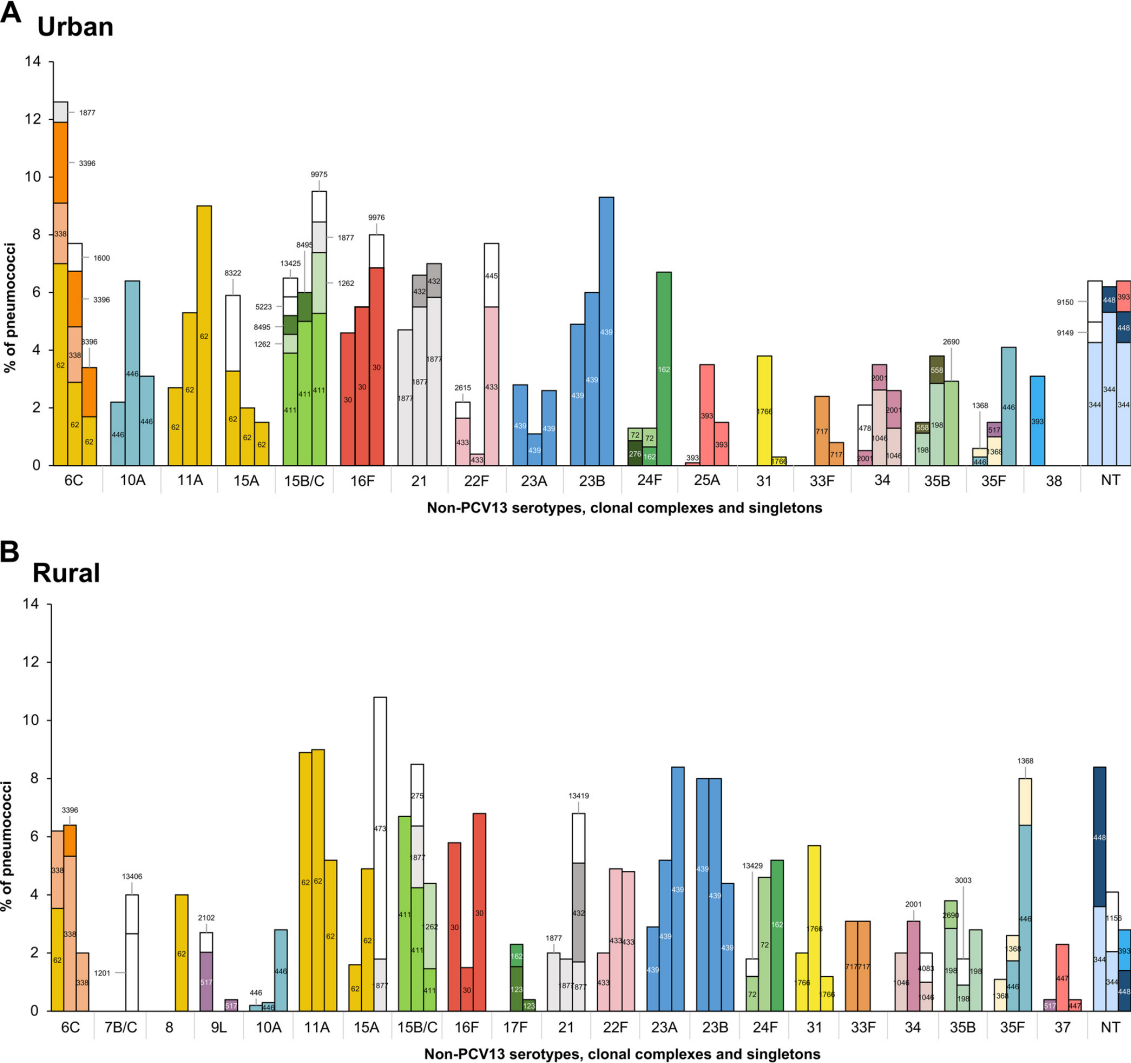
Clonal evolution of the most prevalent non-PCV13 serotypes over time in (A) the urban region and (B) the rural region. Each CC is shown in a different color, and singletons are depicted in white. The numbers inside the bars indicate the founding ST of each CC, as determined using goeBURST. The *y* axis represents the serotype-specific prevalence among pneumococcal carriers. For each serotype, the bars correspond to the pre-, early, and late PCV13 periods, from left to right, respectively. The following non-PCV13 serotypes are not represented here due to sporadic detection: 7A, 7B/C, 9L, 9N, 12A, 12F, 17F, 18A, 20, 25A, 29, 31, 33F, 35A, 37, and 38.

The introduction of PCV13 led to serotype replacement, resulting in a net increase in the prevalence of non-PCV13 serotypes (4). However, at the individual level, trends varied across serotypes (4). We investigated which changes occurred among lineages associated with non-PCV13 serotypes following PCV13 use. We observed that in general, the lineages expanding following the introduction of PCV13 were essentially the same already in circulation in the urban and rural regions before PCV13 use (Fig. 3 and Table S4). Exceptions were infrequent and included the detection of novel lineages, which became prevalent among serotypes 22F (CC445), 24F (CC162), 35B (singleton 2690), and 35F (CC446) in the urban region (Fig. 3A and Table S4) and serotypes 8 (CC62), 15A (CC473), 15B/C (CC1262), 21 (CC432), and 24F (CC162) in the rural region (Fig. 3B and Table S4).

### Antimicrobial susceptibility and association with MLST lineages.

We observed that most PCV13 serotype isolates (55%; 11/20) in the late PCV13 period were resistant to one or more antimicrobials (Table 1). This contrasted with the pre-PCV13 and early PCV13 periods, during which the corresponding proportions were 38% (31/82) and 44% (21/48), respectively, suggesting that for PCV13 serotypes, the selection of antimicrobial-resistant STs might be occurring (Table S2).

**TABLE 1:** Clonality of antimicrobial-nonsusceptible PCV13 serotypes and resistance profiles^*a*^

Serotype	ST^*b*^	CC	No. of isolates^*c*^	No. of resistant isolates^*d*^	Antimicrobial resistance profile^*e*^					
					Urban			Rural		
					Pre-PCV13	Early PCV13	Late PCV13	Pre-PCV13	Early PCV13	Late PCV13
6A	5847		1	1				S		
6B	469	338	2	2	E (1)	E (1)				
14	15		1	1	P, E, C, S					
	143	162	1	1	P					
	156	162	5	5		P, S (1)	P, S (1)	P, S (1)	P, S (2)	
	4575	162	3	3		P, E, S				
18C	**13409**	123	1	1						S
19A	276	276	11	11	P, E, C, T (6)	P, E, C, T (1)		P, E, C, T (3)		P, E, C, T, S (1)
	**13424**	276	1	1	P, E, T					
	320		4	4	P, E, C, T, S (2)	P, E, C, T, S (2)				
	416	411	1	1	E, C, T					
	9151	411	2	2	E, C, T					
	193	1877	2	2	E, C, T, Ch (1)		E, C, T (1)			
	809	1877	1	1			E, C, T			
19F	179	179	17	16	E, C, T (5)	E, C, T (2)	E, C, T (1)	E, C, T (1)	E, C, T (3)	E, C, T (4)
	9721	179	1	1					E, C, T	
	271		3	3	P, E, C, S (1)	P, E, C, S (2)				
	9716		1	1		S				
	9725		1	1	S					
23F	63		1	1	P, E, C, T					
	277	338	2	2		P, S				
	338	338	1	1			P, S			
	**13451**	338	1	1	P					

a ST, sequence type; CC, clonal complex.

b New STs are highlighted in bold.

c Number of isolates typed using MLST.

d Number of resistant isolates among the isolates typed using MLST.

e Antimicrobials to which resistance was detected: S, sulfamethoxazole-trimethoprim; E, erythromycin; P, penicillin; C, clindamycin; T, tetracycline; Ch, chloramphenicol. Numbers in parentheses indicate the number of isolates with that resistance profile.

On the other hand, for non-PCV13 serotypes, there was no clear evidence of selection of antimicrobial-resistant STs after PCV13 introduction (Table 2). While the emergence of resistance was detected in some STs (mostly sporadic isolates of ST386-6C, ST374-15A, ST193-15B/C, ST7006-16F, ST162-24F, ST13415-24F, ST717-33F, ST1046-34, ST1619-NT, and ST13418-NT in the urban region and ST989-12F, ST162-24F, and ST8967-34 in the rural region), most (84%; 139/153) non-PCV13 isolates circulating in the late PCV13 period were susceptible to all antimicrobials tested (Table S2). Indeed, among 128 STs associated with non-PCV13 serotypes, only 44 were associated with antimicrobial resistance (Table 2; Table S2).

**TABLE 2:** Clonality of antimicrobial-nonsusceptible non-PCV13 serotypes and resistance profiles^*a*^

Serotype	ST^*b*^	CC	No. of isolates^*c*^	No. of resistant isolates^*d*^	Antimicrobial resistance profile^*e*^					
					Urban			Rural		
					Pre-PCV13	Early PCV13	Late PCV13	Pre-PCV13	Early PCV13	Late PCV13
6C	1150	338	4	1				P		
	2777	338	2	1					P	
	386	3396	2	1			P, E, C, T			
	3396	3396	6	6	P, E, C, T (4)	E, C, T (1)			E, C, T (1)	
	**13436**	3396	1	1		E, C, T				
10A	97	446	8	4		E, C				
	**13426**	446	1	1	E, C					
11A	62	62	21	1		S				
	408	62	13	2	S (1)			S (1)		
12F	989		1	1						T, Ch
15A	63	62	11	10	P, E, C (2)	P, E, C (1)	P, E, C, T (1)	P, E, C (2)	P, E, C (4)	
	374	62	1	1			P, E, C, T			
	2105	62	2	2	P, E, C, T					
	3816	62	1	1		P, E, C, T				
	**13427**	62	1	1	E, C					
15B/C	275		2	2					P	
	411	411	23	1		P				
	1262		5	3	S (1)		S (2)			
	193	1877	3	1			E, C, T			
	5223		1	1	S					
	8495		2	2	S (1)	S (1)				
	**13425**		1	1	S					
16F	7006	30	2	2		E, C, T, Ch (1)	E, C, T (1)			
24F	162	162	8	8		S (1)	S (4)			S (3)
	13415	162	1	1			S			
	230	276	2	2	P, E, C, T					
	**13429**		1	1				E		
33F	717		12	12		E, C, T (3)	E, C, T (2)	E, C (4)	E, C (3)	
34	1046	1046	5	5		S (2)	S (1)	S (2)		
	8967	1046	1	1						S
	**13443**	1046	1	1		S				
35A	3214		1	1				E, T, S		
35B	198		12	1				P		
	558		2	2	P (1)	P (1)				
NT	344	344	11	11	P, E, C, T, S (2)	P, E, C, T, S (3)	E, C, T, S (1)	P, E, C, T, S (3)	P, E, C, T, S (2)	
	897	344	1	1		E, C, T, S				
	1619	344	1	1			P, E, C, T, S			
	3097	344	5	5	P, E, C, T, S (3)	P, E, T, S (1)	E, C, T, S (1)			
	4149	344	1	1		P, E, C, T, S				
	9722	344	1	1	P, E, C, T, S					
	**13418**	344	1	1			P, E, C, S			
	1156		2	2		P, E, C, T, S				
	9149		1	1	P, S					

a ST, sequence type; CC, clonal complex; NT, nontypeable pneumococci.

b New STs are highlighted in bold.

c Number of isolates typed using MLST.

d Number of resistant isolates among the isolates typed using MLST.

e Antimicrobials to which resistance was detected: S, sulfamethoxazole-trimethoprim; E, erythromycin; P, penicillin; C, clindamycin; T, tetracycline; Ch, chloramphenicol. Numbers in parentheses indicate the number of isolates with that resistance profile.

### Potential capsular switch events.

We identified 13 cases in which a common ST was associated with more than one serotype, suggesting that a capsular switch event had occurred at some point in time (Fig. 1). Of these, seven cases had been observed earlier, in studies conducted in Portugal, or were already described in the MLST database, suggesting that these events predated our study: ST63-15A/23F, ST193-15(B/C)/19A/21, ST191-7A/7F, ST199-15(B/C)/19A, ST393-25A/38/NT, ST439-23A/23B, and ST1201-7(B/C)/19A (Fig. 1). Of these, the first two included multidrug-resistant isolates; the other cases represented fully susceptible isolates.

The other six cases included associations of a given ST with one or more serotypes not described before, suggesting that it was a recent capsular switch event: ST30-12F (most isolates were of serotype 16F), ST66-37 (associated with serotype 9L), ST447-19A (associated with serotype 37), ST1877-6C/19A (associated with serotype 21), ST13422-18A (associated with serotype 15B/C), and ST13423-12A (associated with serotype 21). Notably, all these novel associations were detected in the pre-PCV13 period, and none of them included an ST previously associated with a vaccine type (Table S4). Furthermore, all isolates were fully susceptible to the antimicrobial agents tested.

There were, in addition, seven other examples in which isolates had STs that were single-locus variants (SLVs) of each other but expressed different capsules (ST460-6A and ST97-10A, ST1876-6A and ST473-15A, ST156-14 and ST162-24F, ST4575-14 and ST162-24F, ST271-19F and ST320-19A, ST276-19A and ST230-24F, and ST338-23F and ST2777-6C; Fig. 1). Of these, all but one pair (the exception being ST271-19F and ST320-19A) included lineages previously associated with a PCV13 serotype but now expressing a non-PCV13 serotype, and all but one pair included antimicrobial-resistant isolates (the exception being ST1876-6A and ST473-15A). Still, only three seemed to be novel, as the non-vaccine type SLVs were first detected after PCV13 introduction (ST460-6A and ST97-10A, ST156-14 and ST162-24F, and ST4575-14 and ST162-24F, all first detected in the early PCV13 period; Fig. 1).

## DISCUSSION

We recently reported that the private use of PCV13 in Portugal led to significant changes in the pneumococcal population carried by children in both an urban region and a rural region (4). We observed that extensive serotype replacement and a general decrease in antimicrobial resistance rates had occurred in both regions. This was due to a significant decrease in the prevalence of PCV13 serotypes, which are often associated with antimicrobial resistance.

In this study, we investigated the clonal composition of the pneumococci carried following the private use of PCV13. This was done by applying MLST to a subset of isolates representing all serotypes detected in each study period and in each region.

We observed high genetic diversity among pneumococci, as reflected in the high number of STs identified. Most CCs and several singletons were found in both regions, reflecting the geographic dissemination of multiple lineages. Several of the lineages in circulation had been described previously and included various PMEN clones that have achieved wide geographic distribution and are epidemiologically and clinically relevant (16–19).

In parallel with a decrease in the prevalence of PCV13 serotypes, we observed a decrease in the number of clonal lineages associated with them. The ones still in circulation in the late PCV13 period were already circulating before the introduction of PCV13. Notably, we reported that PCV13 serotypes were more frequently isolated from nonvaccinated children (4). The reasons for the selective persistence of some lineages are not known but may be associated with their antimicrobial resistance profiles, which confer an advantage, given the selective pressure exerted by antimicrobial consumption. This was the case, for example, for the most frequent PCV13 serotype detected in the late PCV13 period, serotype 19F, mainly associated with CC179 (PMEN clone Portugal^19F^-177). Isolates of this lineage are frequently multidrug resistant, showing nonsusceptibility to penicillin and resistance to macrolides, lincosamides, streptogramins, and tetracyclines (16, 17). Interestingly, all serotype 3 isolates in circulation after the introduction of PCV13 were susceptible to antimicrobials and belonged to ST180 (PMEN Netherlands^3^-180). This clone has been detected in children in Portugal since the pre-PCV7 era (10) and remains in circulation in several countries, despite the use of PCV13 (recently reviewed by Azarian et al. [20]). In Portugal, serotype 3 is an important cause of invasive pneumococcal disease (IPD) in both children and adults (14, 21).

We also observed that the increase in the prevalence of non-PCV13 serotypes (due to serotype replacement) was accompanied by the expansion of lineages already in circulation. The emergence of novel clones, while also detected, had less impact. In contrast with clones associated with PCV13 serotypes, in the late PCV13 period, clones associated with non-PCV13 serotypes were often susceptible to most antimicrobial agents. These observations suggest that the selective pressure imposed by PCV13 was, by 2015 to 2016, higher than that imposed by antimicrobial use.

Of major importance was the emergence of ST53 associated with serotype 8 (also known as PMEN clone Netherlands^8^-53), which has high invasive disease potential (22) and is a major cause of adult IPD, in Portugal (21).

While we observed various examples of the same ST being associated with different capsular types, there was no evidence that capsular switching had occurred as a consequence of PCV13 use. On the other hand, this phenomenon appeared to be relatively frequent, involving various serotypes, regardless of whether they were targeted by PCV13. Still, after PCV13 introduction, an expansion of the lineages originally expressing PCV13 vaccine types but now expressing non-vaccine types (SLVs of the original) were observed with increasing frequency. These often included antimicrobial-resistant isolates. Whole-genome sequencing (WGS) analysis could provide additional information on the genetic relationships of these isolates, as well as on the genomic regions affected by recombination, apart from the capsular loci, following horizontal gene transfer.

Our study has some limitations. Only a subset of all isolates were tested, albeit representative of all serotypes detected in all study periods of both regions. In addition, we did not use WGS, which might have been more informative and enabled direct comparison with important ongoing surveillance initiatives, such as the Global Pneumococcal Sequencing Project (available at https://www.pneumogen.net). This will be the focus of future studies.

Our study also has some strengths. First, this is the only study in Portugal reporting the impact of PCV13 on the evolution of pneumococcal clones carried by children attending day care centers. Second, we analyzed a great number of samples using the state-of-the-art technique for molecular typing of S. pneumoniae, MLST. Third, the samples are from two geographical regions, one urban and one rural, and from three periods, representing not only the years after PCV13 introduction but also a baseline that reflects the epidemiological scenario immediately prior to vaccine introduction.

In conclusion, the extensive serotype replacement observed in carriage following the private use of PCV13 was mainly due to the expansion of non-PCV13 clones previously in circulation in Portugal. The emergence of non-PCV13 clones occurred to a lesser extent. Maintenance of PCV13-associated clones was often associated with antimicrobial resistance, except for ST180 associated with serotype 3. There was no evidence of capsular switch events triggered by PCV13 use. However, the expansion of lineages previously associated with PCV13 serotypes but now expressing non-vaccine types was observed. These results not only elucidate the changes that occurred in the pneumococcal population following PCV13 private use but will also be important for understanding the impact of its inclusion in the Portuguese NIP.

## MATERIALS AND METHODS

### Study design and pneumococcal isolates.

Sample collection, isolation of pneumococci, and serotyping were performed as previously described, and the results were recently reported (4). Briefly, pneumococcal carriage isolates (*n* = 2,615) were collected in six cross-sectional studies conducted between 2009 and 2016, in two regions of Portugal (Oeiras and Montemor-o-Novo), from nasopharyngeal swabs of children up to 6 years of age attending day care centers (DCCs). Three time periods were defined: 2009 to 2010 (pre-PCV13), 2011 to 2012 (early PCV13), and 2015 to 2016 (late PCV13). Pure cultures of pneumococci were isolated using gentamicin blood agar. Isolates were serotyped by conventional multiplex PCR following the CDC protocols; the Quellung reaction was used for those isolates for which a serotype could not be assigned by PCR (23, 24).

### Antimicrobial susceptibility testing.

Antimicrobial susceptibility results were reported previously (4). Briefly, susceptibility to chloramphenicol, clindamycin, erythromycin, oxacillin, sulfamethoxazole/trimethoprim, and tetracycline (Oxoid, Hampshire, England) was determined using the disk diffusion method. MICs to amoxicillin, ceftriaxone, and penicillin (oral penicillin V) were determined using the Etest (bioMérieux, Marcy l’Etoile, France). The results were interpreted following CLSI guidelines (25). For penicillin, the CLSI breakpoints for oral penicillin V were used: isolates with an MIC of 0.12 to 1 μg/mL were considered intermediately resistant; isolates with an MIC of ≥2 μg/mL were considered resistant. Multidrug resistance was defined as nonsusceptibility to three or more classes of antimicrobial agents.

### Multilocus sequence typing.

MLST was carried out in the current study for selected isolates: for each time period, and each region, a minimum of 20% of the isolates of each serotype were randomly chosen. In total, out of 2,618 isolates, 657 were typed using MLST: 271 from the pre-PCV13 period, 213 from the early PCV7 period, and 173 from the late PCV13 period (see Table S1 in the supplemental material). The isolates were plated onto tryptic soy agar (TSA) plates supplemented with 5% sheep blood and incubated overnight at 37°C in a 5% CO_2_ atmosphere. On the following day, a loopful (10 μL) of pneumococci was resuspended in 500 μL of TE buffer (10 mM Tris-HCl, 1 mM EDTA, pH 8) and heated for 10 min at 95°C. A 1:10 dilution of boiled cells with sterilized water was used as the DNA template. PCR amplification of internal fragments of seven housekeeping genes (*aroE*, *gdh*, *gki*, *recP*, *spi*, *xpt*, and *ddl*) was performed using previously described primers (26). The final concentrations of the reagents used in the PCR were as follows: 1× GoTaq Flexi Buffer, 2 mM MgCl_2_, 0.04 mM deoxynucleoside triphosphates (dNTPs), 0.4 pmol/μL forward primer, 0.4 pmol/μL reverse primer, and 0.025 U/μL GoTaq; 2 μL of the template DNA was added to a final volume of 20 μL. PCR was carried out as follows: initial denaturation at 94°C for 4 min; 30 cycles of denaturation at 94°C for 30 s, annealing (temperature dependent on the primer used) for 30 s, and elongation at 72°C for 30 s; and a final elongation at 72°C for 10 min. The sequencing reactions were conducted by STABVIDA (Caparica, Portugal) or Macrogen (Amsterdam, The Netherlands). The DNA sequences were analyzed using BioNumerics v6.6 (Applied Maths, Sint-Martens-Latem, Belgium) and DNAStar v7.0.0 (Lasergene) software. Alleles and sequence types (STs) were assigned in accordance with the MLST database for S. pneumoniae available at http://pubmlst.org/spneumoniae/.

Novel alleles and STs were submitted to the MLST database. Whenever the same ST was found to be associated with more than one serotype, suggesting a capsular switch event, MLST and serotyping were repeated.

The relationships between STs were analyzed using PHYLOViZ v2.0 software with the goeBURST v.2.0.2 algorithm (27, 28). STs were represented as nodes, and those that were related up to double-locus variants (DLVs) were joined, forming a tree descriptive of the clonal complexes (CCs). The CC was named after the founder ST. The founder ST was identified as the ST with the higher number of SLVs and DLVs (27, 28). STs that had allelic profiles that could not be joined, at the SLV or DLV level, with other STs were identified as singletons.

The overall prevalence of each CC, per serotype, for each time period and region, was estimated by multiplying the number of isolates of that serotype/period/region, typed by MLST with that CC, by the total number of isolates with that serotype/period/region.
