# Supplementary material for: Clonal Changes in the Pneumococcal Population Carried by Portuguese Children during Six Years of Private Use of the 13-Valent Pneumococcal Conjugate Vaccine: the Relative Contribution of Clonal Expansion, Clonal Emergence, and Capsular Switch Events
Source: Microbiol Spectr. 2023 Mar 22;11(2):e02909-22. doi: 10.1128/spectrum.02909-22 (PMC10100364; doi:10.1128/spectrum.02909-22)
Supplement: Supplemental file 1 — Supplemental material. Download spectrum.02909-22-s0001.pdf, PDF file, 0.3 MB [file spectrum.02909-22-s0001.pdf]

Table S1. Selection of pneumococcal isolates genotyped by MLST

Serotypes	No. of pneumococcal isolates typed by MLST[1]/total no. of isolates[2]						
	Pre-PCV13		Early-PCV13		Late-PCV13		Total
	Urban	Rural	Urban	Rural	Urban	Rural	
**PCV13 serotypes**							
**1**	1/2	0/0	1/2	0/0	0/0	0/0	2/4
**3**	15/68	4/18	5/20	6/26	1/3	2/6	33/141
**5**	0/0	2/6	0/0	0/0	0/0	0/0	2/6
**6A**	3/10	2/9	3/10	2/2	1/2	0/0	11/33
**6B**	1/2	1/3	2/6	0/0	1/5	0/0	5/16
**7F**	1/3	4/15	1/1	2/6	0/0	0/0	8/25
**14**	2/6	1/4	4/16	2/9	1/2	0/0	10/37
**18C**	0/0	0/0	0/0	0/0	0/0	1/1	1/1
**19A**	18/95	11/50	5/20	3/14	2/5	1/2	40/186
**19F**	11/48	3/12	6/27	4/14	3/11	5/19	32/131
**23F**	2/3	0/0	2/7	0/0	2/9	0/0	6/19
**Non-PCV13 serotypes**							
**6C**	18/85	7/30	8/36	6/25	4/13	1/5	44/194
**7A**	0/0	1/5	0/0	0/0	0/0	0/0	1/5
**7B/C**	0/0	0/0	0/0	0/0	1/1	3/10	4/11
**8**	0/0	0/0	0/0	0/0	1/3	3/10	4/13
**9L**	0/0	4/12	0/0	0/0	1/1	1/1	6/14
**9N**	0/0	1/1	1/2	0/0	0/0	0/0	2/3
**10A**	3/15	1/1	7/30	1/1	3/12	3/7	18/66
**11A**	4/18	9/41	6/24	8/35	8/35	3/13	38/166
**12A**	0/0	1/1	0/0	0/0	0/0	0/0	1/1
**12F**	1/2	0/0	0/0	0/0	0/0	1/1	2/3
**15A**	9/40	2/7	2/9	4/19	2/6	6/27	25/108
**15B/C**	10/45	7/32	6/27	8/35	9/37	3/11	43/187
**16F**	7/31	6/26	6/25	2/6	7/31	4/17	32/136
**17F**	0/0	0/0	1/1	3/9	1/1	1/1	6/12
**18A**	0/0	1/2	0/0	0/0	0/0	0/0	1/2
**20**	1/1	0/0	1/2	0/0	1/1	0/0	3/4
**21**	8/35	3/9	6/30	2/7	6/27	4/17	29/125
**22F**	4/15	3/9	1/2	5/19	7/30	3/12	23/87
**23A**	4/18	4/13	1/5	5/20	3/10	5/21	22/87
**23B**	7/33	8/36	6/29	7/31	8/36	3/11	39/176
**24F**	3/9	3/9	2/6	4/18	5/26	4/13	21/81
**25A**	1/1	0/0	4/16	0/0	2/6	1/2	8/25
**29**	0/0	0/0	2/7	0/0	0/0	0/0	2/7
**31**	0/0	2/9	4/17	6/22	1/1	2/3	15/52
**33F**	0/0	4/14	3/11	3/12	2/3	0/0	12/40
**34**	4/14	2/9	4/16	3/12	2/10	2/5	17/66
**35A**	0/0	1/2	0/0	0/0	0/0	0/0	1/2
**35B**	4/10	4/17	4/17	2/7	4/15	2/7	20/73
**35F**	2/4	1/5	3/8	3/10	4/16	5/20	18/63
**37**	0/0	1/2	0/0	2/9	2/5	1/1	6/17
**38**	5/21	2/7	0/0	2/6	0/0	0/0	9/34
**NT**	9/43	7/37	7/28	4/16	6/25	2/7	35/156
**Total**	**23.3%** (158/677)	**24.9%** (113/453)	**24.9%** (114/457)	**25.8%** (99/390)	**25.8%** (101/388)	**28.8%** (72/250)	**25.1%** (657/2615)

[1]At least 20% of the isolates of each serotype, year and location, chosen randomly.

[2]Serotypes and antibiotypes of these isolates were described previously.

NT, nontypeable pneumococci

Table S2. Region, period of isolation, serotype and genotype of pneumococcal isolates.

Isolate	Region	Period	Serotype	ST[1]	CC/Singleton[2]	Resistance profile[3]
6354	Rural	Pre-PCV13	19A	1201	1201*	-
6360	Rural	Pre-PCV13	19A	276	276	P,E,C,T
6368	Rural	Pre-PCV13	19A	276	276	P,E,C,T
6390	Rural	Pre-PCV13	NT	344	344	P,E,C,T,S
6409	Rural	Pre-PCV13	NT	344	344	P,E,C,T,S
6432	Rural	Pre-PCV13	15A	63	62	P,E,C
6433	Rural	Pre-PCV13	33F	717	717*	E,C
6435	Rural	Pre-PCV13	24F	72	72*	-
6438	Rural	Pre-PCV13	9L	66	517	-
6439	Rural	Pre-PCV13	NT	448	448*	-
6442	Rural	Pre-PCV13	35B	198	198*	-
6449	Rural	Pre-PCV13	35B	198	198*	-
6466	Rural	Pre-PCV13	5	1223	1223*	-
6471	Rural	Pre-PCV13	16F	30	30	-
6472	Rural	Pre-PCV13	5	1223	1223*	-
6477	Rural	Pre-PCV13	16F	30	30	-
6485	Rural	Pre-PCV13	19F	177	179	-
6489	Rural	Pre-PCV13	6C	395	62	-
6495	Rural	Pre-PCV13	19A	276	276	P,E,C,T
6522	Rural	Pre-PCV13	7A	191	191*	-
6533	Rural	Pre-PCV13	15B/C	411	411	-
6536	Rural	Pre-PCV13	15B/C	411	411	-
6538	Rural	Pre-PCV13	11A	62	62	-
6560	Rural	Pre-PCV13	6A	5847	5847*	S
6576	Rural	Pre-PCV13	33F	717	717*	E,C
6579	Rural	Pre-PCV13	37	66	517	-
6580	Rural	Pre-PCV13	19A	447	447*	-
6586	Rural	Pre-PCV13	19A	3017	3017*	-
6592	Rural	Pre-PCV13	24F	72	72*	-
6597	Rural	Pre-PCV13	9L	2102	2102*	-
6600	Rural	Pre-PCV13	21	1877	1877	-
6602	Rural	Pre-PCV13	15B/C	411	411	-
6612	Rural	Pre-PCV13	9L	66	517	-
6626	Rural	Pre-PCV13	22F	4334	433	-
6628	Rural	Pre-PCV13	23A	8866	439	-
6637	Rural	Pre-PCV13	18A	**13422**	411	-
6643	Rural	Pre-PCV13	15B/C	**13422**	411	-
6654	Rural	Pre-PCV13	31	1766	1766*	-
6660	Rural	Pre-PCV13	35F	1368	1368*	-
6676	Rural	Pre-PCV13	12A	**13423**	1877	-
6679	Rural	Pre-PCV13	21	**13423**	1877	-
6687	Rural	Pre-PCV13	35B	198	198*	P
6690	Rural	Pre-PCV13	NT	344	344	P,E,C,T,S
6695	Rural	Pre-PCV13	11A	408	62	S
6704	Rural	Pre-PCV13	6C	1150	338	P
6706	Rural	Pre-PCV13	7F	191	191*	-

Table S2. (cont.)

Isolate	Region	Period	Serotype	ST[1]	CC/Singleton[2]	Resistance profile[3]
6707	Rural	Pre-PCV13	6C	1150	338	-
6708	Rural	Pre-PCV13	6C	1150	338	-
6709	Rural	Pre-PCV13	23B	439	439	-
6712	Rural	Pre-PCV13	23B	439	439	-
6713	Rural	Pre-PCV13	7F	191	191*	-
6717	Rural	Pre-PCV13	11A	408	62	-
6719	Rural	Pre-PCV13	15B/C	411	411	-
6726	Urban	Pre-PCV13	35F	**13548**	446	-
6735	Urban	Pre-PCV13	23A	439	439	-
6744	Urban	Pre-PCV13	15B/C	8495	8495*	S
6749	Urban	Pre-PCV13	19A	**13424**	276	P,E,T
6759	Urban	Pre-PCV13	3	180	180	-
6784	Urban	Pre-PCV13	15A	2105	62	P,E,C,T
6801	Urban	Pre-PCV13	19A	276	276	P,E,C,T
6808	Urban	Pre-PCV13	19A	276	276	P,E,C,T
6812	Urban	Pre-PCV13	15B/C	411	411	-
6815	Urban	Pre-PCV13	19F	179	179	E,C,T
6833	Urban	Pre-PCV13	23B	439	439	-
6836	Urban	Pre-PCV13	NT	344	344	P,E,C,T,S
6842	Urban	Pre-PCV13	21	1877	1877	-
6852	Urban	Pre-PCV13	7F	191	191*	-
6854	Urban	Pre-PCV13	38	393	393*	-
6860	Urban	Pre-PCV13	38	393	393*	-
6867	Urban	Pre-PCV13	19A	1151	1151*	-
6877	Urban	Pre-PCV13	3	180	180	-
6890	Urban	Pre-PCV13	19A	1151	1151*	-
6893	Urban	Pre-PCV13	6C	3396	3396	P,E,C,T
6904	Urban	Pre-PCV13	3	180	180	-
6906	Urban	Pre-PCV13	6C	395	62	-
6910	Urban	Pre-PCV13	11A	62	62	-
6912	Urban	Pre-PCV13	19A	1877	1877	-
6922	Urban	Pre-PCV13	19A	276	276	P,E,C,T
6943	Urban	Pre-PCV13	19F	179	179	E,C,T
6944	Urban	Pre-PCV13	1	306	306*	-
6970	Urban	Pre-PCV13	38	393	393*	-
6975	Urban	Pre-PCV13	6C	395	62	-
6988	Urban	Pre-PCV13	21	1877	1877	-
6992	Urban	Pre-PCV13	19A	320	320*	P,E,C,T,S
6994	Urban	Pre-PCV13	16F	30	30	-
7012	Urban	Pre-PCV13	3	180	180	-
7015	Urban	Pre-PCV13	15B/C	411	411	-
7017	Urban	Pre-PCV13	3	180	180	-
7024	Urban	Pre-PCV13	15B/C	411	411	-
7031	Urban	Pre-PCV13	NT	344	344	P,E,C,T,S
7040	Urban	Pre-PCV13	15B/C	5223	5223*	S
7042	Urban	Pre-PCV13	6C	395	62	-

Table S2. (cont.)

Isolate	Region	Period	Serotype	ST[1]	CC/Singleton[2]	Resistance profile[3]
7052	Urban	Pre-PCV13	6C	3396	3396	P,E,C,T
7065	Urban	Pre-PCV13	22F	433	433	-
7067	Urban	Pre-PCV13	22F	433	433	-
7071	Urban	Pre-PCV13	3	180	180	-
7076	Urban	Pre-PCV13	6C	3396	3396	P,E,C,T
7077	Urban	Pre-PCV13	14	15	15*	P,E,C,T,S
7098	Urban	Pre-PCV13	3	180	180	-
7102	Urban	Pre-PCV13	3	180	180	-
7104	Urban	Pre-PCV13	19F	9148	179	-
7107	Urban	Pre-PCV13	35B	198	198*	-
7111	Urban	Pre-PCV13	22F	433	433	-
7113	Urban	Pre-PCV13	3	180	180	-
7117	Urban	Pre-PCV13	24F	230	276	P,E,C,T,
7125	Urban	Pre-PCV13	23A	439	439	-
7128	Urban	Pre-PCV13	24F	230	276	P,E,C,T
7144	Urban	Pre-PCV13	3	180	180	-
7148	Urban	Pre-PCV13	23F	63	62	P,E,C,T
7159	Urban	Pre-PCV13	6A	65	446	-
7164	Urban	Pre-PCV13	19F	179	179	E,C,T
7169	Urban	Pre-PCV13	21	1877	1877	-
7170	Urban	Pre-PCV13	16F	30	30	-
7195	Urban	Pre-PCV13	6A	65	446	-
7208	Urban	Pre-PCV13	23A	42	439	-
7229	Urban	Pre-PCV13	3	180	180	-
7234	Urban	Pre-PCV13	19A	193	1877	Ch,E,C,T
7235	Urban	Pre-PCV13	15B/C	1262	1262*	S
7236	Urban	Pre-PCV13	3	180	180	-
7244	Urban	Pre-PCV13	15A	8322	8322*	-
7248	Urban	Pre-PCV13	NT	3097	344	P,E,C,T,S
7249	Urban	Pre-PCV13	NT	3097	344	P,E,C,T,S
7250	Urban	Pre-PCV13	19A	276	276	P,E,C,T
7252	Urban	Pre-PCV13	6C	1877	1877	-
7256	Urban	Pre-PCV13	19F	9725	9725*	S
7259	Urban	Pre-PCV13	19A	276	276	P,E,C,T
7266	Urban	Pre-PCV13	NT	3097	344	P,E,C,T,S
7270	Urban	Pre-PCV13	15B/C	411	411	-
7271	Urban	Pre-PCV13	15B/C	**13425**	13425*	S
7272	Urban	Pre-PCV13	NT	9149	9149*	P,S
7280	Urban	Pre-PCV13	15A	63	62	P,E,C
7287	Urban	Pre-PCV13	15A	63	62	P,E,C
7308	Urban	Pre-PCV13	19F	177	179	-
7318	Urban	Pre-PCV13	11A	408	62	S
7325	Urban	Pre-PCV13	11A	408	62	-
7326	Urban	Pre-PCV13	23B	439	439	-
7327	Urban	Pre-PCV13	34	**13450**	2001	-
7338	Urban	Pre-PCV13	21	1877	1877	-

Table S2. (cont.)

Isolate	Region	Period	Serotype	ST[1]	CC/Singleton[2]	Resistance profile[3]
7343	Urban	Pre-PCV13	19A	994	994*	-
7353	Urban	Pre-PCV13	19A	276	276	P,E,C,T
7355	Urban	Pre-PCV13	23B	439	439	-
7369	Urban	Pre-PCV13	6C	3396	3396	P,E,C,T
7372	Urban	Pre-PCV13	3	180	180	-
7380	Urban	Pre-PCV13	6C	395	62	-
7386	Urban	Pre-PCV13	15B/C	411	411	-
7389	Urban	Pre-PCV13	21	1877	1877	-
7403	Urban	Pre-PCV13	14	143	162	P
7404	Urban	Pre-PCV13	NT	9722	344	P,E,C,T,S
7409	Urban	Pre-PCV13	10A	461	446	-
7414	Urban	Pre-PCV13	10A	461	446	-
7416	Urban	Pre-PCV13	19F	9148	179	-
7418	Urban	Pre-PCV13	15B/C	199	411	-
7429	Urban	Pre-PCV13	NT	9150	9150*	-
7445	Urban	Pre-PCV13	24F	72	72*	-
7468	Urban	Pre-PCV13	6C	2689	338	-
7471	Urban	Pre-PCV13	19A	320	320*	P,E,C,T,S
7473	Urban	Pre-PCV13	23F	**13451**	338	P
7476	Urban	Pre-PCV13	NT	9150	9150*	-
7486	Urban	Pre-PCV13	10A	**13426**	446	E,C
7487	Urban	Pre-PCV13	6C	2689	338	-
7488	Urban	Pre-PCV13	6C	2689	338	-
7503	Urban	Pre-PCV13	19F	9717	179	-
7509	Urban	Pre-PCV13	23B	439	439	-
7514	Urban	Pre-PCV13	16F	30	30	-
7519	Urban	Pre-PCV13	23B	439	439	-
7529	Urban	Pre-PCV13	19F	179	179	E,C,T
7536	Urban	Pre-PCV13	23B	439	439	-
7551	Urban	Pre-PCV13	19A	1151	1151*	-
7575	Urban	Pre-PCV13	6A	65	446	-
7576	Urban	Pre-PCV13	16F	30	30	-
7586	Urban	Pre-PCV13	23B	439	439	-
7599	Urban	Pre-PCV13	34	478	478*	-
7620	Urban	Pre-PCV13	6C	395	62	-
7623	Urban	Pre-PCV13	34	478	478*	-
7625	Urban	Pre-PCV13	34	478	478*	-
7629	Urban	Pre-PCV13	19A	9151	411	E,C,T
7630	Urban	Pre-PCV13	19A	9151	411	E,C,T
7632	Urban	Pre-PCV13	15A	8322	8322*	-
7636	Urban	Pre-PCV13	11A	408	62	-
7637	Urban	Pre-PCV13	35B	558	558*	P
7648	Urban	Pre-PCV13	6C	395	62	-
7651	Urban	Pre-PCV13	6C	395	62	-
7653	Urban	Pre-PCV13	6B	469	338	E
7688	Urban	Pre-PCV13	19A	416	411	E,C,T

Table S2. (cont.)

Isolate	Region	Period	Serotype	ST[1]	CC/Singleton[2]	Resistance profile[3]
7691	Urban	Pre-PCV13	38	393	393*	-
7699	Urban	Pre-PCV13	15A	8322	8322*	-
7704	Urban	Pre-PCV13	6C	395	62	-
7707	Urban	Pre-PCV13	25A	393	393*	-
7710	Urban	Pre-PCV13	15A	2105	62	P,E,C,T
7711	Urban	Pre-PCV13	35B	198	198*	-
7716	Urban	Pre-PCV13	3	180	180	-
7719	Urban	Pre-PCV13	35B	198	198*	-
7721	Urban	Pre-PCV13	15A	8322	8322*	-
7723	Urban	Pre-PCV13	20	1026	1026*	-
7726	Urban	Pre-PCV13	6C	1692	62	-
7727	Urban	Pre-PCV13	19F	179	179	E,C,T
7732	Urban	Pre-PCV13	15A	**13427**	62	E,C
7733	Urban	Pre-PCV13	38	393	393*	-
7754	Urban	Pre-PCV13	6C	395	62	-
7759	Urban	Pre-PCV13	22F	2615	2615*	-
7765	Urban	Pre-PCV13	21	1877	1877	-
7770	Urban	Pre-PCV13	35F	1368	1368*	-
7775	Urban	Pre-PCV13	16F	30	30	-
7776	Urban	Pre-PCV13	12F	30	30	-
7777	Urban	Pre-PCV13	16F	30	30	-
7789	Urban	Pre-PCV13	23A	42	439	-
7792	Urban	Pre-PCV13	16F	30	30	-
7796	Urban	Pre-PCV13	3	180	180	-
7802	Urban	Pre-PCV13	21	1877	1877	-
7804	Urban	Pre-PCV13	21	1877	1877	-
7814	Urban	Pre-PCV13	19F	271	271*	P,E,C,S
7815	Rural	Pre-PCV13	9N	66	517	-
7833	Rural	Pre-PCV13	34	1046	1046	S
7834	Rural	Pre-PCV13	23B	439	439	-
7837	Rural	Pre-PCV13	19A	1201	1201*	-
7839	Rural	Pre-PCV13	19A	1201	1201*	-
7841	Rural	Pre-PCV13	14	156	162	P,S
7864	Rural	Pre-PCV13	34	1046	1046	S
7889	Rural	Pre-PCV13	23A	190	439	-
7891	Rural	Pre-PCV13	23B	**13428**	439	-
7903	Rural	Pre-PCV13	35B	2690	2690*	-
7905	Rural	Pre-PCV13	21	13423	1877	-
7913	Rural	Pre-PCV13	11A	9724	62	-
7920	Rural	Pre-PCV13	11A	62	62	-
7922	Rural	Pre-PCV13	11A	62	62	-
7929	Rural	Pre-PCV13	16F	30	30	-
7931	Rural	Pre-PCV13	16F	30	30	-
7933	Rural	Pre-PCV13	11A	62	62	-
7951	Rural	Pre-PCV13	16F	30	30	-
7959	Rural	Pre-PCV13	38	393	393*	-

Table S2. (cont.)

Isolate	Region	Period	Serotype	ST[1]	CC/Singleton[2]	Resistance profile[3]
7965	Rural	Pre-PCV13	6C	395	62	-
7974	Rural	Pre-PCV13	7F	191	191*	-
7976	Rural	Pre-PCV13	38	393	393*	-
7979	Rural	Pre-PCV13	7F	191	191*	-
7985	Rural	Pre-PCV13	6C	395	62	-
7993	Rural	Pre-PCV13	22F	433	433	-
8001	Rural	Pre-PCV13	35A	3214	3214*	E,T,S
8003	Rural	Pre-PCV13	3	180	180	-
8004	Rural	Pre-PCV13	6C	395	62	-
8008	Rural	Pre-PCV13	3	180	180	-
8010	Rural	Pre-PCV13	10A	461	446	-
8020	Rural	Pre-PCV13	33F	717	717*	E,C
8033	Rural	Pre-PCV13	15A	63	62	P,E,C
8036	Rural	Pre-PCV13	33F	717	717*	E,C
8037	Rural	Pre-PCV13	15B/C	411	411	-
8043	Rural	Pre-PCV13	19A	9160	9160*	-
8046	Rural	Pre-PCV13	22F	9161	433	-
8049	Rural	Pre-PCV13	23B	9155	439	-
8058	Rural	Pre-PCV13	3	9162	180	-
8060	Rural	Pre-PCV13	24F	**13429**	13429*	E
8069	Rural	Pre-PCV13	16F	30	30	-
8074	Rural	Pre-PCV13	11A	62	62	-
8077	Rural	Pre-PCV13	23A	42	439	-
8088	Rural	Pre-PCV13	15B/C	411	411	-
8092	Rural	Pre-PCV13	19A	1201	1201*	-
8097	Rural	Pre-PCV13	3	180	180	-
8098	Rural	Pre-PCV13	6B	9164	9164*	-
8100	Rural	Pre-PCV13	23B	439	439	-
8108	Rural	Pre-PCV13	23B	439	439	-
8124	Rural	Pre-PCV13	11A	62	62	-
8135	Rural	Pre-PCV13	31	1766	1766*	-
8148	Rural	Pre-PCV13	19F	177	179	-
8151	Rural	Pre-PCV13	23A	42	439	-
8163	Rural	Pre-PCV13	9L	517	517	-
8166	Rural	Pre-PCV13	NT	448	448*	-
8167	Rural	Pre-PCV13	NT	448	448*	-
8169	Rural	Pre-PCV13	NT	448	448*	-
8172	Rural	Pre-PCV13	23B	8722	439	-
8180	Rural	Pre-PCV13	19F	179	179	E,C,T
8184	Rural	Pre-PCV13	6A	1876	1876*	-
8185	Rural	Pre-PCV13	19A	994	994*	-
8191	Urban	Early-PCV13	6C	2689	338	-
8207	Urban	Early-PCV13	10A	97	446	E,C
8208	Urban	Early-PCV13	11A	408	62	-
8209	Urban	Early-PCV13	6C	2689	338	-
8210	Urban	Early-PCV13	19A	199	411	-

Table S2. (cont.)

Isolate	Region	Period	Serotype	ST[1]	CC/Singleton[2]	Resistance profile[3]
8215	Urban	Early-PCV13	6A	460	446	-
8227	Urban	Early-PCV13	7F	191	191*	-
8231	Urban	Early-PCV13	21	432	432*	-
8251	Urban	Early-PCV13	3	180	180	-
8261	Urban	Early-PCV13	23B	439	439	-
8270	Urban	Early-PCV13	21	193	1877	-
8281	Urban	Early-PCV13	6A	65	446	-
8292	Urban	Early-PCV13	35F	1368	1368*	-
8311	Urban	Early-PCV13	10A	461	446	-
8314	Urban	Early-PCV13	19A	994	994*	-
8338	Urban	Early-PCV13	3	1220	1220*	-
8341	Urban	Early-PCV13	33F	717	717*	E,C,T
8343	Urban	Early-PCV13	15A	63	62	P,E,C
8352	Urban	Early-PCV13	16F	30	30	-
8361	Urban	Early-PCV13	23F	277	338	P,S
8363	Urban	Early-PCV13	14	4575	162	P,E,S
8365	Urban	Early-PCV13	19F	9716	9716*	S
8366	Urban	Early-PCV13	31	1766	1766*	-
8367	Urban	Early-PCV13	19F	179	179	E,C,T
8374	Urban	Early-PCV13	31	1766	1766*	-
8404	Urban	Early-PCV13	31	1766	1766*	-
8408	Urban	Early-PCV13	11A	408	62	-
8416	Urban	Early-PCV13	10A	97	446	E,C
8420	Urban	Early-PCV13	14	4575	162	P,E,S
8421	Urban	Early-PCV13	33F	717	717*	E,C,T
8429	Urban	Early-PCV13	NT	897	344	E,C,T,S
8431	Urban	Early-PCV13	24F	72	72*	-
8432	Urban	Early-PCV13	19F	179	179	E,C,T
8436	Urban	Early-PCV13	21	1877	1877	-
8444	Urban	Early-PCV13	10A	461	446	-
8445	Urban	Early-PCV13	11A	62	62	S
8455	Urban	Early-PCV13	25A	393	393*	-
8459	Urban	Early-PCV13	NT	3097	344	P,E,T,S
8466	Urban	Early-PCV13	25A	393	393*	-
8469	Urban	Early-PCV13	34	**13430**	2001	-
8470	Urban	Early-PCV13	25A	393	393*	-
8472	Urban	Early-PCV13	21	1877	1877	-
8495	Urban	Early-PCV13	6B	469	338	E
8497	Urban	Early-PCV13	25A	393	393*	-
8504	Urban	Early-PCV13	11A	408	62	-
8511	Urban	Early-PCV13	35B	558	558*	P
8512	Urban	Early-PCV13	16F	30	30	-
8513	Urban	Early-PCV13	23A	438	439	-
8520	Urban	Early-PCV13	15B/C	411	411	-
8527	Urban	Early-PCV13	19F	271	271*	P,E,C,S
8529	Urban	Early-PCV13	19A	320	320*	P,E,C,T,S

Table S2. (cont.)

Isolate	Region	Period	Serotype	ST[1]	CC/Singleton[2]	Resistance profile[3]
8532	Urban	Early-PCV13	6C	395	62	-
8534	Urban	Early-PCV13	19F	271	271*	P,E,C,S
8542	Urban	Early-PCV13	31	1766	1766*	-
8545	Urban	Early-PCV13	16F	7006	30	Ch,E,C,T
8561	Urban	Early-PCV13	19A	320	320*	P,E,C,T,S
8568	Urban	Early-PCV13	15B/C	8495	8495*	S
8569	Urban	Early-PCV13	NT	4149	344	P,E,C,T,S
8575	Urban	Early-PCV13	23B	439	439	-
8582	Urban	Early-PCV13	35F	1368	1368*	-
8591	Urban	Early-PCV13	15B/C	411	411	P
8599	Urban	Early-PCV13	6C	395	62	-
8607	Urban	Early-PCV13	10A	97	446	-
8610	Urban	Early-PCV13	16F	30	30	-
8613	Urban	Early-PCV13	34	1046	1046	S
8622	Urban	Early-PCV13	21	1877	1877	-
8633	Urban	Early-PCV13	15B/C	411	411	-
8635	Urban	Early-PCV13	6C	3396	3396	E,C,T
8636	Urban	Early-PCV13	20	1026	1026*	-
8651	Rural	Early-PCV13	7F	9719	9719*	-
8654	Rural	Early-PCV13	19F	179	179	E,C,T
8667	Rural	Early-PCV13	15B/C	275	275*	P
8669	Rural	Early-PCV13	19F	179	179	E,C,T
8676	Rural	Early-PCV13	15B/C	411	411	-
8680	Rural	Early-PCV13	17F	392	162	-
8686	Rural	Early-PCV13	22F	433	433	-
8689	Rural	Early-PCV13	23B	439	439	-
8690	Rural	Early-PCV13	6C	3396	3396	E,C,T
8701	Rural	Early-PCV13	3	180	180	-
8712	Rural	Early-PCV13	11A	62	62	-
8723	Rural	Early-PCV13	NT	344	344	P,E,C,T,S
8729	Rural	Early-PCV13	6C	1150	338	-
8732	Rural	Early-PCV13	23B	9155	439	-
8736	Rural	Early-PCV13	14	156	162	P,S
8748	Rural	Early-PCV13	38	393	393*	-
8754	Rural	Early-PCV13	22F	433	433	-
8760	Rural	Early-PCV13	31	1766	1766*	-
8770	Rural	Early-PCV13	33F	717	717*	E,C
8773	Rural	Early-PCV13	24F	72	72*	-
8777	Rural	Early-PCV13	23A	42	439	-
8790	Rural	Early-PCV13	24F	72	72*	-
8792	Rural	Early-PCV13	19A	1151	1151*	-
8801	Rural	Early-PCV13	33F	717	717*	E,C
8812	Rural	Early-PCV13	35F	446	446	-
8815	Rural	Early-PCV13	3	180	180	-
8819	Rural	Early-PCV13	14	156	162	P,S
8820	Rural	Early-PCV13	16F	30	30	-

Table S2. (cont.)

Isolate	Region	Period	Serotype	ST[1]	CC/Singleton[2]	Resistance profile[3]
8824	Rural	Early-PCV13	34	2001	2001	-
8825	Rural	Early-PCV13	11A	62	62	-
8826	Rural	Early-PCV13	19A	1201	1201*	-
8828	Rural	Early-PCV13	3	1220	1220*	-
8855	Rural	Early-PCV13	11A	62	62	-
8859	Rural	Early-PCV13	37	447	447*	-
8865	Rural	Early-PCV13	23B	439	439	-
8878	Rural	Early-PCV13	6C	2689	338	-
8880	Rural	Early-PCV13	11A	62	62	-
8889	Rural	Early-PCV13	23A	42	439	-
8898	Rural	Early-PCV13	NT	1156	1156*	P,E,C,T,S
8910	Rural	Early-PCV13	24F	72	72*	-
8911	Rural	Early-PCV13	11A	62	62	-
8912	Rural	Early-PCV13	23A	42	439	-
8917	Rural	Early-PCV13	35B	198	198*	-
8926	Rural	Early-PCV13	11A	62	62	-
8937	Rural	Early-PCV13	22F	433	433	-
8938	Rural	Early-PCV13	35F	1368	1368*	-
8951	Rural	Early-PCV13	17F	4002	123	-
8959	Rural	Early-PCV13	23A	190	439	-
8966	Rural	Early-PCV13	6A	1876	1876*	-
8970	Rural	Early-PCV13	21	1877	1877	-
8977	Rural	Early-PCV13	33F	717	717*	E,C
8980	Rural	Early-PCV13	16F	**13433**	30	-
8987	Rural	Early-PCV13	11A	62	62	-
8992	Rural	Early-PCV13	15B/C	411	411	-
8994	Rural	Early-PCV13	34	**13452**	2001	-
9001	Rural	Early-PCV13	19A	1201	1201*	-
9014	Rural	Early-PCV13	23B	439	439	-
9019	Rural	Early-PCV13	3	180	180	-
9022	Rural	Early-PCV13	34	**13452**	2001	-
9025	Rural	Early-PCV13	10A	461	446	-
9048	Rural	Early-PCV13	23B	439	439	-
9057	Rural	Early-PCV13	23B	439	439	-
9060	Rural	Early-PCV13	15B/C	275	275*	P
9061	Rural	Early-PCV13	6A	460	446	-
9069	Rural	Early-PCV13	15B/C	411	411	-
9075	Rural	Early-PCV13	15A	63	62	P,E,C
9078	Rural	Early-PCV13	7F	191	191*	-
9080	Rural	Early-PCV13	31	1766	1766*	-
9085	Rural	Early-PCV13	6C	2689	338	-
9091	Rural	Early-PCV13	21	**13434**	1877	-
9092	Rural	Early-PCV13	38	393	393*	-
9100	Rural	Early-PCV13	15A	63	62	P,E,C
9108	Rural	Early-PCV13	15A	63	62	P,E,C
9109	Rural	Early-PCV13	NT	1156	1156*	P,E,C,T,S

Table S2. (cont.)

Isolate	Region	Period	Serotype	ST[1]	CC/Singleton[2]	Resistance profile[3]
9114	Rural	Early-PCV13	19F	179	179	E,C,T
9117	Rural	Early-PCV13	6C	2777	338	-
9123	Rural	Early-PCV13	23B	439	439	-
9126	Rural	Early-PCV13	6C	2777	338	P
9136	Rural	Early-PCV13	15B/C	411	411	-
9143	Rural	Early-PCV13	17F	4002	123	-
9150	Rural	Early-PCV13	31	1766	1766*	-
9154	Rural	Early-PCV13	11A	408	62	-
9159	Rural	Early-PCV13	22F	433	433	-
9171	Rural	Early-PCV13	31	1766	1766*	-
9174	Rural	Early-PCV13	15B/C	193	1877	-
9176	Rural	Early-PCV13	22F	433	433	-
9177	Rural	Early-PCV13	19F	9721	179	E,C,T
9194	Rural	Early-PCV13	23A	42	439	-
9200	Rural	Early-PCV13	37	447	447*	-
9204	Rural	Early-PCV13	15B/C	193	1877	-
9208	Rural	Early-PCV13	31	1766	1766*	-
9210	Rural	Early-PCV13	35F	446	446	-
9229	Rural	Early-PCV13	24F	72	72*	-
9239	Rural	Early-PCV13	15A	63	62	P,E,C
9241	Rural	Early-PCV13	31	1766	1766*	-
9243	Rural	Early-PCV13	3	9723	180	-
9244	Rural	Early-PCV13	NT	344	344	P,E,C,T,S
9248	Rural	Early-PCV13	35B	3003	3003*	-
9258	Rural	Early-PCV13	3	180	180	-
9266	Urban	Early-PCV13	23B	439	439	-
9271	Urban	Early-PCV13	33F	717	717*	E,C,T
9275	Urban	Early-PCV13	6C	1692	62	-
9282	Urban	Early-PCV13	17F	392	162	-
9284	Urban	Early-PCV13	3	180	180	-
9300	Urban	Early-PCV13	15B/C	411	411	-
9305	Urban	Early-PCV13	34	1046	1046	S
9311	Urban	Early-PCV13	23B	439	439	-
9318	Urban	Early-PCV13	15B/C	**13435**	411	-
9329	Urban	Early-PCV13	3	180	180	-
9347	Urban	Early-PCV13	6C	**13436**	3396	E,C,T
9348	Urban	Early-PCV13	NT	344	344	P,E,C,T,S
9350	Urban	Early-PCV13	35B	198	198*	-
9354	Urban	Early-PCV13	11A	**13438**	62	-
9355	Urban	Early-PCV13	NT	344	344	P,E,C,T,S
9364	Urban	Early-PCV13	35F	2975	517	-
9372	Urban	Early-PCV13	15A	3816	62	P,E,C,T
9376	Urban	Early-PCV13	23B	439	439	-
9381	Urban	Early-PCV13	35B	198	198*	-
9385	Urban	Early-PCV13	19F	9717	179	-
9392	Urban	Early-PCV13	23B	439	439	-

Table S2. (cont.)

Isolate	Region	Period	Serotype	ST[1]	CC/Singleton[2]	Resistance profile[3]
9395	Urban	Early-PCV13	35B	198	198*	-
9396	Urban	Early-PCV13	14	156	162	P,S
9407	Urban	Early-PCV13	21	**13439**	1877	-
9410	Urban	Early-PCV13	14	4575	162	P,E,S
9414	Urban	Early-PCV13	29	**13441**	13441*	-
9423	Urban	Early-PCV13	10A	97	446	E,C
9441	Urban	Early-PCV13	NT	344	344	P,E,C,T,S
9442	Urban	Early-PCV13	6C	1600	1600*	-
9448	Urban	Early-PCV13	6B	176	338	-
9451	Urban	Early-PCV13	10A	97	446	E,C
9455	Urban	Early-PCV13	23F	277	338	P,S
9477	Urban	Early-PCV13	6A	65	446	-
9482	Urban	Early-PCV13	29	**13442**	13442*	-
9492	Urban	Early-PCV13	19A	276	276	P,E,C,T
9494	Urban	Early-PCV13	24F	162	162	S
9506	Urban	Early-PCV13	22F	433	433	-
9520	Urban	Early-PCV13	1	306	306*	-
9527	Urban	Early-PCV13	34	**13443**	1046	S
9528	Urban	Early-PCV13	3	180	180	-
9529	Urban	Early-PCV13	16F	30	30	-
9536	Urban	Early-PCV13	9N	66	517	-
9538	Urban	Early-PCV13	NT	448	448*	-
9539	Urban	Early-PCV13	11A	408	62	-
9550	Urban	Early-PCV13	16F	30	30	-
10261	Rural	Late-PCV13	23A	42	439	-
10273	Rural	Late-PCV13	24F	162	162	S
10285	Rural	Late-PCV13	19A	276	276	P,E,C,T,S
10288	Rural	Late-PCV13	16F	30	30	-
10295	Rural	Late-PCV13	22F	433	433	-
10307	Rural	Late-PCV13	23A	42	439	-
10311	Rural	Late-PCV13	11A	62	62	-
10316	Rural	Late-PCV13	12F	989	989*	Ch,T
10321	Rural	Late-PCV13	24F	162	162	S
10332	Rural	Late-PCV13	16F	30	30	-
10343	Rural	Late-PCV13	24F	162	162	S
10345	Rural	Late-PCV13	15A	473	473*	-
10353	Rural	Late-PCV13	19F	179	179	E,C,T
10365	Rural	Late-PCV13	8	53	62	-
10367	Rural	Late-PCV13	19F	179	179	E,C,T
10369	Rural	Late-PCV13	21	432	432*	-
10370	Rural	Late-PCV13	23B	439	439	-
10374	Rural	Late-PCV13	NT	448	448*	-
10379	Rural	Late-PCV13	10A	97	446	-
10380	Rural	Late-PCV13	7B/C	**13406**	13406*	-
10383	Rural	Late-PCV13	7B/C	1201	1201*	-
10387	Rural	Late-PCV13	15A	5139	1877	-

Table S2. (cont.)

Isolate	Region	Period	Serotype	ST[1]	CC/Singleton[2]	Resistance profile[3]
10391	Rural	Late-PCV13	6C	2689	338	-
10402	Rural	Late-PCV13	7B/C	1201	1201*	-
10407	Rural	Late-PCV13	15B/C	411	411	-
10418	Rural	Late-PCV13	17F	123	123	-
10426	Rural	Late-PCV13	35B	198	198*	-
10427	Rural	Late-PCV13	31	1766	1766*	-
10441	Rural	Late-PCV13	22F	433	433	-
10449	Rural	Late-PCV13	35F	1635	446	-
10450	Rural	Late-PCV13	35F	**13407**	446	-
10453	Rural	Late-PCV13	21	**13408**	1877	-
10455	Rural	Late-PCV13	37	447	447*	-
10457	Rural	Late-PCV13	18C	**13409**	123	S
10458	Rural	Late-PCV13	3	9071	180	-
10461	Rural	Late-PCV13	11A	**13410**	62	-
10470	Rural	Late-PCV13	15B/C	1262	1262*	-
10476	Rural	Late-PCV13	15A	473	473*	-
10481	Rural	Late-PCV13	34	8967	1046	S
10489	Urban	Late-PCV13	15B/C	411	411	-
10496	Urban	Late-PCV13	15B/C	411	411	-
10502	Urban	Late-PCV13	21	1877	1877	-
10519	Urban	Late-PCV13	16F	30	30	-
10522	Urban	Late-PCV13	23F	338	338	P,S
10527	Urban	Late-PCV13	11A	62	62	-
10528	Urban	Late-PCV13	16F	30	30	-
10537	Urban	Late-PCV13	15B/C	411	411	-
10542	Urban	Late-PCV13	7B/C	1201	1201*	-
10559	Urban	Late-PCV13	21	1877	1877	-
10569	Urban	Late-PCV13	11A	408	62	-
10584	Urban	Late-PCV13	35B	2690	2690*	-
10587	Urban	Late-PCV13	35B	2690	2690*	-
10597	Urban	Late-PCV13	6C	386	3396	-
10600	Urban	Late-PCV13	35F	1635	446	-
10604	Urban	Late-PCV13	35B	452	452*	-
10613	Urban	Late-PCV13	35F	1635	446	-
10624	Urban	Late-PCV13	15B/C	199	411	-
10626	Urban	Late-PCV13	34	1046	1046	S
10628	Urban	Late-PCV13	19F	179	179	E,C,T
10632	Urban	Late-PCV13	37	**13412**	13412*	-
10640	Urban	Late-PCV13	35F	1635	446	-
10641	Urban	Late-PCV13	15B/C	1262	1262*	S
10647	Urban	Late-PCV13	22F	10220	433	-
10648	Urban	Late-PCV13	NT	344	344	E,C,T,S
10651	Urban	Late-PCV13	NT	1619	344	P,E,C,T,S
10657	Urban	Late-PCV13	15A	374	62	P,E,C,T
10659	Urban	Late-PCV13	19F	391	179	-
10661	Urban	Late-PCV13	10A	97	446	-

Table S2. (cont.)

Isolate	Region	Period	Serotype	ST[1]	CC/Singleton[2]	Resistance profile[3]
10667	Urban	Late-PCV13	11A	62	62	-
10675	Urban	Late-PCV13	6B	176	338	-
10682	Urban	Late-PCV13	20	1026	1026*	-
10698	Urban	Late-PCV13	22F	433	433	-
10706	Urban	Late-PCV13	22F	433	433	-
10708	Urban	Late-PCV13	23A	42	439	-
10714	Urban	Late-PCV13	23B	439	439	-
10722	Urban	Late-PCV13	6C	1692	62	-
10724	Urban	Late-PCV13	6C	**13413**	62	-
10726	Urban	Late-PCV13	33F	717	717*	E,C,T
10727	Urban	Late-PCV13	22F	445	445*	-
10730	Urban	Late-PCV13	22F	445	445*	-
10731	Urban	Late-PCV13	16F	30	30	-
10738	Urban	Late-PCV13	16F	30	30	-
10762	Urban	Late-PCV13	17F	11176	162	-
10768	Urban	Late-PCV13	23B	439	439	-
10775	Urban	Late-PCV13	16F	7006	30	E,C,T
10781	Urban	Late-PCV13	19A	809	1877	E,C,T
10785	Urban	Late-PCV13	10A	461	446	-
10786	Urban	Late-PCV13	23B	**13414**	439	-
10795	Urban	Late-PCV13	24F	**13415**	162	S
10809	Urban	Late-PCV13	15A	63	62	P,E,C,T
10815	Urban	Late-PCV13	10A	97	446	-
10818	Urban	Late-PCV13	22F	**13416**	433	-
10836	Urban	Late-PCV13	23A	42	439	-
10849	Urban	Late-PCV13	23B	10039	439	-
10857	Urban	Late-PCV13	23B	10039	439	-
10862	Urban	Late-PCV13	23B	439	439	-
10867	Urban	Late-PCV13	21	1877	1877	-
10876	Urban	Late-PCV13	23B	**13417**	439	-
10879	Urban	Late-PCV13	21	1877	1877	-
10881	Urban	Late-PCV13	23B	10039	439	-
10892	Urban	Late-PCV13	34	2001	2001	-
10893	Urban	Late-PCV13	19A	193	1877	E,C,T
10897	Urban	Late-PCV13	23F	12464	30	-
10898	Urban	Late-PCV13	6C	386	3396	P,E,C,T
10917	Urban	Late-PCV13	15B/C	9975	9975*	-
10922	Urban	Late-PCV13	NT	393	393*	-
10927	Urban	Late-PCV13	9L	66	517	-
10936	Urban	Late-PCV13	25A	393	393*	-
10937	Urban	Late-PCV13	22F	433	433	-
10939	Urban	Late-PCV13	6A	65	446	-
10941	Urban	Late-PCV13	25A	393	393*	-
10949	Urban	Late-PCV13	23A	438	439	-
10959	Urban	Late-PCV13	16F	30	30	-
10964	Urban	Late-PCV13	NT	**13418**	344	P,E,C,S

Table S2. (cont.)

Isolate	Region	Period	Serotype	ST[1]	CC/Singleton[2]	Resistance profile[3]
10975	Urban	Late-PCV13	31	1766	1766*	T,S
10977	Urban	Late-PCV13	35F	1635	446	-
10981	Urban	Late-PCV13	11A	62	62	-
10987	Urban	Late-PCV13	15B/C	1262	1262*	S
10989	Urban	Late-PCV13	NT	3097	344	E,C,T,S
10994	Urban	Late-PCV13	24F	162	162	S
10998	Urban	Late-PCV13	37	447	447*	-
11020	Urban	Late-PCV13	11A	62	62	-
11021	Urban	Late-PCV13	11A	62	62	-
11032	Urban	Late-PCV13	21	1877	1877	-
11034	Urban	Late-PCV13	24F	162	162	S
11045	Urban	Late-PCV13	21	432	432*	-
11063	Urban	Late-PCV13	14	156	162	P,S
11064	Urban	Late-PCV13	33F	717	717*	E,C,T
11070	Urban	Late-PCV13	19F	179	179	-
11076	Urban	Late-PCV13	16F	9976	9976*	-
11085	Urban	Late-PCV13	15B/C	193	1877	E,C,T
11086	Urban	Late-PCV13	35B	2690	2690*	-
11088	Urban	Late-PCV13	11A	408	62	-
11092	Urban	Late-PCV13	11A	408	62	-
11106	Urban	Late-PCV13	3	180	180	-
11110	Urban	Late-PCV13	24F	162	162	S
11111	Urban	Late-PCV13	15B/C	2220	411	-
11112	Urban	Late-PCV13	8	53	62	-
11115	Urban	Late-PCV13	24F	162	162	S
11123	Rural	Late-PCV13	10A	461	446	-
11134	Rural	Late-PCV13	16F	30	30	-
11137	Rural	Late-PCV13	10A	461	446	-
11148	Rural	Late-PCV13	16F	30	30	-
11154	Rural	Late-PCV13	21	**13419**	13419*	-
11169	Rural	Late-PCV13	25A	393	393*	-
11171	Rural	Late-PCV13	23B	439	439	-
11173	Rural	Late-PCV13	19F	179	179	E,C,T
11175	Rural	Late-PCV13	15A	473	473*	-
11188	Rural	Late-PCV13	35F	446	446	-
11191	Rural	Late-PCV13	23B	439	439	-
11193	Rural	Late-PCV13	NT	393	393*	-
11222	Rural	Late-PCV13	19F	**13420**	179	-
11223	Rural	Late-PCV13	24F	72	72*	-
11226	Rural	Late-PCV13	31	1766	1766*	-
11232	Rural	Late-PCV13	15B/C	1262	1262*	-
11243	Rural	Late-PCV13	8	53	62	-
11252	Rural	Late-PCV13	23A	42	439	-
11261	Rural	Late-PCV13	35F	1368	1368*	-
11263	Rural	Late-PCV13	22F	433	433	-
11265	Rural	Late-PCV13	19F	179	179	E,C,T

Table S2. (cont.)

Isolate	Region	Period	Serotype	ST[1]	CC/Singleton[2]	Resistance profile[3]
11266	Rural	Late-PCV13	23A	**13549**	439	-
11272	Rural	Late-PCV13	23A	**13449**	439	-
11278	Rural	Late-PCV13	3	180	180	-
11280	Rural	Late-PCV13	11A	**13421**	62	-
11284	Rural	Late-PCV13	15A	473	473*	-
11287	Rural	Late-PCV13	15A	473	473*	-
11290	Rural	Late-PCV13	35B	198	198*	-
11293	Rural	Late-PCV13	21	432	432*	-
11294	Rural	Late-PCV13	34	4083	4083*	-
11306	Rural	Late-PCV13	8	53	62	-
11308	Rural	Late-PCV13	35F	446	446	-
11315	Rural	Late-PCV13	9L	66	517	-
11319	Urban	Late-PCV13	NT	448	448*	-

[1]Novel STs are highlighted in bold.
[2]Singletons are indicated with an asterisk.
[3]Antimicrobials for which resistance was detected: S, sulfamethoxazole-trimethoprim; E, erythromycin; P, penicillin; C, clindamycin; T, tetracycline; Ch, chloramphenicol.
ST, sequence type; CC, clonal complex; NT, nontypeable pneumococci.

Table S3. Clonal evolution of PCV13 serotypes

PCV13 serotypes	ST (no. of isolates)					
	Urban			Rural		
	Pre-PCV13	Early-PCV13	Late-PCV13	Pre-PCV13	Early-PCV13	Late-PCV13
**1**	306	306	-	-	-	-
**3**	180 (15)	180 (4)<br>1220	180	180 (3)<br>9162	180 (4)<br>1220<br>9723	180<br>9071
**5**	-	-	-	1223 (2)	-	-
**6A**	65 (3)	65 (2)<br>460	65	1876<br>5847	1876<br>460	-
**6B**	469	469<br>176	176	9164	-	-
**7F**	191	191	-	191 (4)	191<br>9719	-
**14**	15<br>143	4575 (3)<br>156	156	156	156 (2)	-
**18C**	-	-	-	-	-	**13409**
**19A**	276 (6)<br>320 (2)<br>994<br>193<br>1877<br>**13424**<br>1151 (3)<br>416<br>9151 (2)	276<br>320 (2)<br>994<br>199	193<br>809	276 (3)<br>994<br>9160<br>1201 (4)<br>447<br>3017	1201 (2)<br>1151	276
**19F**	179 (5)<br>177<br>9717<br>9148 (2)<br>271<br>9725	179 (2)<br>9717<br>271 (2)<br>9716	179 (2)<br>391	179<br>177 (2)	179 (3)<br>9721	179 (4)<br>**13420**
**23F**	63<br>**13451**	277 (2)	338<br>12464	-	-	-

New STs are highlighted in bold. Underlined STs indicate isolates resistant to at least one of the following antimicrobial agents: penicillin, chloramphenicol, erythromycin, clindamycin, tetracycline, and sulfamethoxazole-trimethoprim. Numbers in parenthesis indicate the number of isolates with that ST. Absence of numbers in parenthesis indicate ST of a single isolate.

Table S4. Clonal evolution of non-PCV13 serotypes

Non-PCV13 serotypes	ST (no. of isolates)					
	Urban			Rural		
	Pre-PCV13	Early-PCV13	Late-PCV13	Pre-PCV13	Early-PCV13	Late-PCV13
**6C**	395 (9)<br>1692<br>2689 (3)<br>1877<br>3396 (4)	395 (2)<br>1692<br>2689 (2)<br>3396<br>**13436**<br>1600	386 (2)<br>1692<br>**13413**	395 (4)<br>1150 (3)	3396<br>1150<br>2689 (2)<br>2777 (2)	2689
**7A**	-	-	-	191	-	-
**7B/C**	-	-	1201	-	-	1201 (2)<br>**13406**
**8**	-	-	53	-	-	53 (3)
**9L**	-	-	66	66 (2)<br>517<br>2102	-	66
**9N**	-	66	-	66	-	-
**10A**	461 (2)<br>**13426**	97 (5)<br>461 (2)	97 (2)<br>461	461	461	461 (2)<br>97
**11A**	408 (3)<br>62	408 (4)<br>62<br>**13438**	408 (3)<br>62 (5)	62 (6)<br>408 (2)<br>9724	62 (7)<br>408	62<br>**13410**<br>**13421**
**12A**	-	-	-	**13423**	-	-
**12F**	30	-	-	-	-	989
**15A**	63 (2)<br>2105 (2)<br>**13427**<br>8322 (4)	63<br>3816	63<br>374	63 (2)	63 (4)	473 (5)<br>5139
**15B/C**	411 (5)<br>199<br>5223<br>8495<br>**13425**<br>1262	411 (4)<br>8495<br>**13435**	411 (3)<br>193<br>199<br>1262 (2)<br>2220<br>9975	411 (6)<br>**13422**	411 (4)<br>275 (2)<br>193 (2)	411<br>1262 (2)
**16F**	30 (7)	30 (5)<br>7006	30 (5)<br>7006<br>9976	30(6)	30<br>**13433**	30 (4)
**17F**	-	392	11176	-	4002 (2)<br>392	123
**18A**	-	-	-	**13422**	-	-
**20**	1026	1026	1026	-	-	-
**21**	1877 (8)	1877 (3)<br>**13439**<br>193<br>432	1877 (5)<br>432	1877<br>**13423 (2)**	1877<br>13434	432 (2)<br>**13408**<br>**13419**
**22F**	433 (3)<br>2615	433	433 (3)<br>445 (2)<br>10220<br>**13416**	433<br>4334<br>9161	433 (5)	433 (3)
**23A**	42 (2)<br>439 (2)	438	42 (2)<br>438	42 (2)<br>190<br>8866	42 (4)<br>190	42 (3)<br>**13449**<br>**13549**

Table S4 (cont.)

Non-PCV13 serotypes	ST (no. of isolates)					
	Urban			Rural		
	Pre-PCV13	Early-PCV13	Late-PCV13	Pre-PCV13	Early-PCV13	Late-PCV13
**23B**	439 (7)	439 (6)	439 (3)<br>10039 (3)<br>**13414**<br>**13417**	439 (5)<br>8722<br>9155<br>**13428**	439 (6)<br>9155	439 (3)
**24F**	230 (2)<br>72	72<br>162	162 (4)<br>**13415**	72 (2)<br>**13429**	72 (4)	72<br>162 (3)
**25A**	393	393 (4)	393 (2)	-	-	393
**29**	-	**13441**<br>**13442**	-	-	-	-
**31**	-	1766 (4)	1766	1766 (2)	1766 (6)	1766 (2)
**33F**	-	717 (3)	717 (2)	717 (4)	717 (3)	-
**34**	478 (3)<br>**13450**	1046 (2)<br>**13430**<br>**13443**	1046<br>2001	1046 (2)	2001<br>**13452 (2)**	8967<br>4083
**35A**	-	-	-	3214	-	-
**35B**	198 (3)<br>558	198 (3)<br>558	2690 (3)<br>452	198 (3)<br>2690	198<br>3003	198 (2)
**35F**	1368<br>**13548**	1368 (2)<br>2975	1635 (4)	1368	446 (2)<br>1368	446 (2)<br>1368<br>1635<br>**13407**
**37**	-	-	447<br>**13412**	66	447 (2)	13411
**38**	393 (5)	-	-	393 (2)	393 (2)	-
**NT**	344 (2)<br>3097 (3)<br>9722<br>9149<br>9150 (2)	344 (3)<br>3097<br>897<br>4149<br>448	344<br>393<br>448<br>1619<br>3097<br>**13418**	344 (3)<br>448 (4)	344 (2)<br>1156 (2)	393<br>448

New STs are highlighted in bold. Underlined STs indicate isolates resistant to at least one of the following antimicrobial agents: penicillin, chloramphenicol, erythromycin, clindamycin, tetracycline, and sulfamethoxazole-trimethoprim. Numbers in parenthesis indicate the number of isolates with that ST. Absence of numbers in parenthesis indicate ST of a single isolate.
